# Health Tract, No. 201

**Published:** 1864-03

**Authors:** 


### HEALTH TRACT, No. 201.

### APOPLEXY

MEANS "stricken from;" a description given by the Greeks, under the feeling that it was of unearthly origin. The person falls down as if suddenly struck with death. There is neither thought, feeling, nor voluntary motion. There is no sign of life, except that of deep heavy breathing. It comes on with the suddenness of the lightning's flash, and with as little premonition. A common fainting fit occurs suddenly, but there is no breathing, no pulse, and the face is pale and shrunken. In apoplexy, if the person is not really dead, the face is flushed, the breathing loud, and the pulse full and strong, usually. In mild attacks, a person is found in bed of a morning apparently in a sound sleep; but if so, he can be easily waked up. In apoplexy no amount of shaking makes any impression. The earliest Greek writers described apoplexy with a minute accuracy, which has scarcely been exceeded since, showing that it is a malady belonging to all time. To pass from apparent perfect health to instant death on entering one's own dwelling, or sitting down to the family table, or while at the happy fireside, in the loving interchange of affectionate offices, strikes us as being perfectly terrible. But the terror belongs to the witnesses; the victim is as perfectly destitute of thought, feeling, sensation, and consciousness, for the time being, as if the head had been taken off by a cannon-ball. In many cases, after lying for hours and even days in a state of perfect insensibility, the patient wakes up as if from an uneasy sleep or dream; but often, as many sadly know, there is no return to life again. The essential nature of the disease seems to be such an excess of blood in the brain that its appropriate vessels or channels can not contain it, and it is "extravasated," let out, upon the substance of the brain itself, and thus arrests the functions of life. Persons with short neck, who are "thick-set," corpulent, are almost the sole actual subjects of apoplexy, when not induced by falls, blows, shocks, and over-doses of certain drugs. Apoplexy is an avoidable disease, except in some cases of accidents, which we can neither foresee nor prevent; it is, essentially, too much blood in the brain. This blood is either sent there too rapidly, or, when there, is detained in some unnatural manner, the essential effect being the same. Whatever "excites the brain" does so by sending an unnatural amount of blood there; such as intense and long thought on one subject, all kinds of liquors; any drink containing alcohol, whether ale, beer, cider, wine, or brandy, excites the brain and endangers apoplexy. So will a hearty meal, especially if alcoholic drinks are taken at the same time; going to bed soon after eating heartily, sleeping on the back, if corpulent, may bring on an attack any night; so will a hot bath, so will a cold bath soon after eating. The ultimate effects of all opiates are to *detain* the blood in the brain, while the things just mentioned send it there in excess. The great preventives are warm feet, regular daily bodily habits, eating nothing later than three o'clock P.M., and the avoidance of opiates, tobacco, and all that can intoxicate. In case of an attack send for a physician. Meanwhile, put the feet in *hot* water, and envelop the head with *cold;* ice is still better. It is safer to live in a hilly than level country, in town than country. Winter is more dangerous than summer. The liability increases rapidly after forty years of age, greatest at sixty, when it gradually diminishes. Statistics seem to show that the most dangerous years are FORTY-EIGHT, FIFTY-EIGHT, SIXTY-SIX, while *forty-six* and *forty-nine* are almost exempt. The well-to-do are more liable than the laboring. Sudden changes of weather promote attacks. Let the liable, especially, live in reference to these well-established facts.

